# Supplementary material for: The genome sequence of the Figwort Sawfly Tenthredo scrophulariae Linnaeus, 1758
Source: Wellcome Open Res. 2024 Nov 5;9:650. [Version 1] doi: 10.12688/wellcomeopenres.23304.1 (PMC11579585; doi:10.12688/wellcomeopenres.23304.1)
Supplement: Peer review history

# Reviewer report: Reviewer response for version 1

**DOI:** 10.21956/wellcomeopenres.25686.r112414

**Published:** 2024-11-20

**Authors:** Marko Prous

**Affiliations:** 1 University of Tartu, Tartu, Estonia

The paper reports chromosome level assembly of a sawfly *Tenthredo scrophulariae* in standard style to the Darwin Tree of Life Consortium. 

 Noticed a few errors, that should be corrected:

 1) correct "Tenthredo prop *l*inqua" to " *Tenthredo propinqua"*

 2) correct "...and the publication of the complete *gene* sequence..." to "...and the publication of the complete *genome* sequence..."

 3) Estimation of HiFi long read coverage cannot be correct. If the genome is  233 Mb and you have 12 Gb of hifi reads, then coverage can be 12000 Mb / 233 Mb = 51.5 , not >100. Illumina coverage looks correct.

 Otherwise looks ok.

Are sufficient details of methods and materials provided to allow replication by others?

Yes

Is the rationale for creating the dataset(s) clearly described?

Yes

Are the datasets clearly presented in a useable and accessible format?

Yes

Are the protocols appropriate and is the work technically sound?

Yes

Reviewer Expertise:

Taxonomy, phylogeny, genomics of sawflies.

I confirm that I have read this submission and believe that I have an appropriate level of expertise to confirm that it is of an acceptable scientific standard.

# Reviewer report: Reviewer response for version 1

**DOI:** 10.21956/wellcomeopenres.25686.r110244

**Published:** 2024-11-16

**Authors:** Shan Gao

**Affiliations:** 1 Nankai University, Tianjin, China

In the manuscript, the authors provided the genome sequence of *Tenthredo scrophulariae* 

 First, many thanks to the authors for their public welfare contribution. However, I still feel compelled to comment on the less professional aspects of the article and kindly request the authors to consider revising them. I strongly suggest these authors to download a professional genome sequence report and use it as a template for their writing.

 1. In the topic, authors should clearly state the complete genome of 'XX', where 'XX' should be a concise and straightforward term. It is recommended to use the English common name of 

 the species instead of its Latin scientific name for most readers.

 2. In the abstract, authors should clearly report key genomic information, including the estimated size of the genome, the number of chromosomes, and the average sequencing coverage. 

 Additionally, it should be indicated whether this represents the first genome sequenced for this genus or family.  If it is not, they should inform readers about the first sequenced genome 

 in that genus or family.

 3. In the abstract, authors should clearly report key genomic information, including the estimated size of the genome, the number of chromosomes, and the average sequencing coverage. 

 Additionally, it should be indicated whether this represents the first genome sequenced for this genus or family.  If it is not, they should inform readers about the first sequenced genome 

 in that genus or family.

 3. In the Methods, the assembly is no well introduced. I think PacBio data was used to obtain the contigs first and then the contigs were further scaffolded using the HiFi.

 4. Genome sequence report is not professional, I suggested the authors to download a professional one for using as a template to write. A xx genome of xx was obtained, its full length?

 how many chromosomes? is the assembly on chromosome level? how many protein-coding genes, non-coding RNAs were annotated?

 5. Please clearly tell readers is it the first genome for a family? or genus? and what is the genome of its closest species for the same family?

 6. GCA ID need be affiliated its database name and internet weblink.

Are sufficient details of methods and materials provided to allow replication by others?

Yes

Is the rationale for creating the dataset(s) clearly described?

Yes

Are the datasets clearly presented in a useable and accessible format?

Yes

Are the protocols appropriate and is the work technically sound?

Yes

Reviewer Expertise:

Bioinformatics, genomics

I confirm that I have read this submission and believe that I have an appropriate level of expertise to confirm that it is of an acceptable scientific standard.
